# Radial Frequency Analysis of Contour Shapes in the Visual Cortex

**DOI:** 10.1371/journal.pcbi.1004719

**Published:** 2016-02-11

**Authors:** Viljami R. Salmela, Linda Henriksson, Simo Vanni

**Affiliations:** 1 Institute of Behavioural Sciences, Division of Cognitive and Neuropsychology, University of Helsinki, Helsinki, Finland; 2 Advanced Magnetic Imaging Centre, Aalto NeuroImaging, Aalto University School of Science, Espoo, Finland; 3 Department of Neuroscience and Biomedical Engineering, Aalto University, Espoo, Finland; 4 Clinical Neurosciences, Neurology, University of Helsinki and Helsinki University Hospital, Helsinki, Finland; Technische Universitat Chemnitz, GERMANY

## Abstract

Cumulative psychophysical evidence suggests that the shape of closed contours is analysed by means of their radial frequency components (RFC). However, neurophysiological evidence for RFC-based representations is still missing. We investigated the representation of radial frequency in the human visual cortex with functional magnetic resonance imaging. We parametrically varied the radial frequency, amplitude and local curvature of contour shapes. The stimuli evoked clear responses across visual areas in the univariate analysis, but the response magnitude did not depend on radial frequency or local curvature. Searchlight-based, multivariate representational similarity analysis revealed RFC specific response patterns in areas V2d, V3d, V3AB, and IPS0. Interestingly, RFC-specific representations were not found in hV4 or LO, traditionally associated with visual shape analysis. The modulation amplitude of the shapes did not affect the responses in any visual area. Local curvature, SF-spectrum and contrast energy related representations were found across visual areas but without similar specificity for visual area that was found for RFC. The results suggest that the radial frequency of a closed contour is one of the cortical shape analysis dimensions, represented in the early and mid-level visual areas.

## Author Summary

Current views suggest that neural representations of the visual environment are built from combinations of basis functions. For low-level visual feature analysis these basis functions are relatively well understood. It is not yet known, however, how primary visual features are combined into higher-level representations of visual objects. Psychophysical evidence suggests that visual shapes are perceived on the basis of the radial frequency components of the shape contour. We investigated whether human visual cortex contains representations of radial frequency components. Our results show that the neural mechanisms that utilize radial frequency are located in the early and intermediate visual areas, and provide further support for the idea of radial frequency based representations in shape perception. This suggests that radial frequency representations might be one link between low-level visual feature analysis and high-level object shape representations.

## Introduction

**Fig 1:**
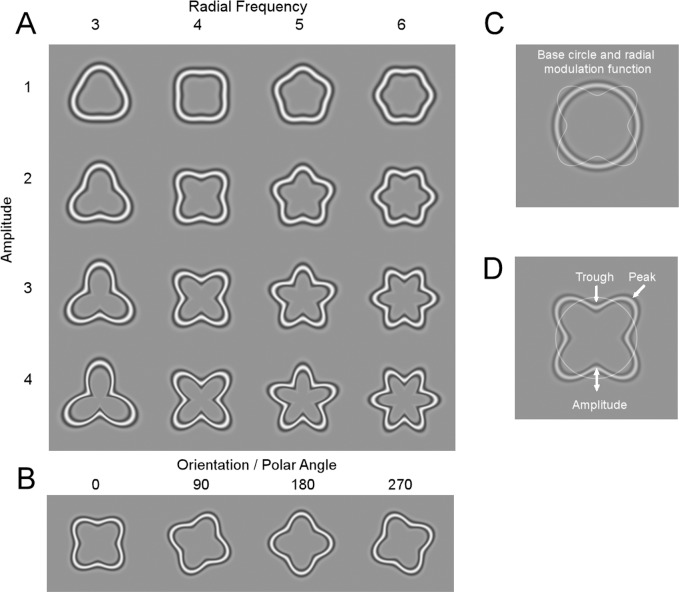
Stimuli. **A)** Radial frequency patterns with different radial frequencies (3–6) and amplitudes (A1-4). **B)** All the different shapes were presented in four different orientations (polar phases 0, 90, 180 or 270 deg). In total, 65 different stimuli were used, circles and 64 modulated shapes (4 RFCs x 4 amplitudes x 4 orientations). **C)** RFC patterns are constructed by modulating a base circle with radial sine function. **D)** The concave and convex curvatures were calculated at the trough and peak, respectively, of the modulation function. Amplitude refers to the amount of modulation relative to the radius of base circle.

To psychophysically investigate contour shape processing beyond local Gabor-like analysis Wilkinson et al. [1] introduced radial frequency patterns (Fig 1A), closed contour shapes formed by sinusoidally modulating the radius of a base circle (Fig 1C). Any closed shape, such as the outline of human face, can be constructed with multiple radial frequency components (RFC) [2]. Wilkinson et al. [1] showed that human observers are extremely sensitive in detecting shape deformation from circularity, with visual acuity exceeding the spatial resolution of the retina. Psychophysical studies have provided converging evidence that visual system relies on global shape analysis of these patterns [1, 3–10]. Experiments using psychophysical methods of adaptation [11, 12], masking [13], and sub-threshold summation [6] suggested that shape analysis of these patterns are RFC specific. This indicates that closed contour shapes are analysed—similarly to local spatial frequency and orientation—via narrow-band radial frequency channels. The neurophysiological evidence for RFC-based shape representations is, however, still missing.

In previous functional magnetic resonance imaging (fMRI) studies, shape representations have been studied with circular gratings and Gabor arrays. Radial and concentric gratings [14] as well as Gabor flow-fields that contain global shape [15] evoke stronger responses in mid-level areas V3 and hV4 than in primary visual cortex (V1) or area V2. Lateral occipital complex (LOC) is also associated with processing of visual objects [16–19] and contours [20]. Human fMRI results are consistent with single-cell recording studies in macaque monkeys that show selectivity for complex shapes in areas V2 [21–24] and V4 [23, 25–29] although complex shape units have been reported also from area V1 [23, 30].

The aim of this work is to test the hypothesis emerging from psychophysical evidence that intermediate shape analysis contains representations of contour RFC. We measured blood oxygenation level dependent (BOLD) responses from the human visual cortex to parametric variation of radial frequency and modulation amplitude of closed contours (Fig 1A). The measured BOLD-responses were analysed with multivariate representational similarity analysis (RSA) [31]. In RSA, correlations between activity patterns evoked by different stimuli are calculated to construct representational dissimilarity matrices (RDMs). To characterize response profiles for different visual areas, the measured RDMs were compared to model RDMs based on stimulus radial frequency, modulation amplitude, local curvature, spatial frequency spectrum and contrast energy. We used a searchlight approach [32] that makes no assumption about location, but instead the whole cortex is scanned voxel-by-voxel to find the stimulus-specific information. We found RFC specific response patterns and our results suggest that mid-level visual areas V2d, V3d, V3AB, and IPS0 contain radial frequency based representations of contour shapes.

## Results

### Attention task

During the measurements, the participants performed a demanding RSVP task at the fixation [18] to control for attention and to ensure that the participant did not attend to any specific shape or part of the contour. The percentage of correct responses varied, both individually and between runs, from 35 to 80%, but was significantly above the chance level of 25% correct (t(10) = 6.124,p < .001). The average of correct responses across participants was 50%.

### BOLD signal changes

**Fig 2:**
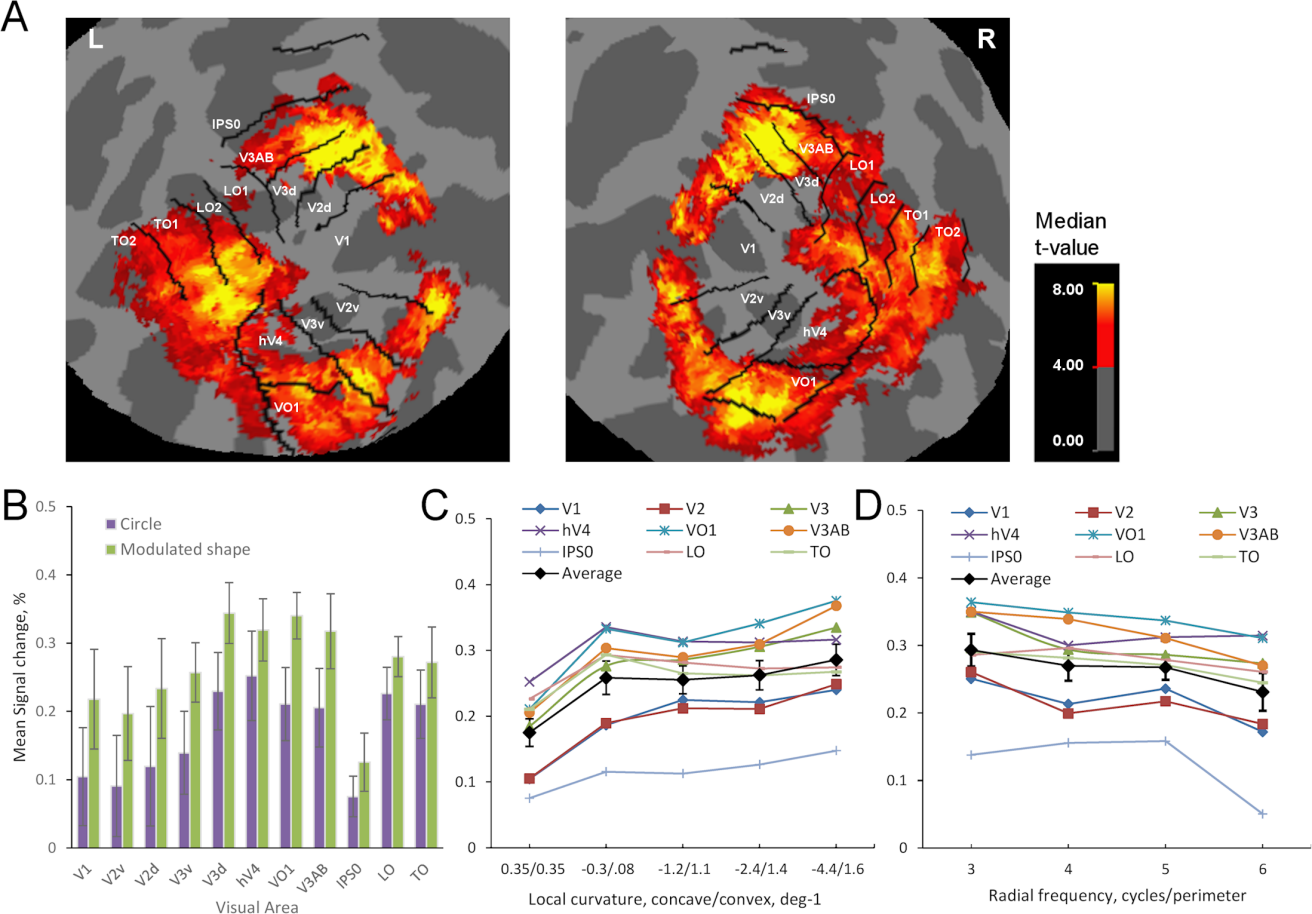
Activity across visual areas. **A)** Within the searchlight, t-value for average activity across different modulated shapes was calculated for each voxel and for each participant. Median t-values across participants are shown on the flattened Freesurfer average surface. Left and right hemispheres are on the left and right sides, respectively. **B)** Signal changes for circles and modulated shapes in different visual areas. **C)** Signal changes as a function of local curvature. Value 0.35 depicts circle shape. **D)** Signal changes as a function of radial frequency in different visual areas.

First we investigated the activity evoked by our shape stimuli by calculating average activity within the searchlight sphere, each with radius of 3 voxels. This analysis is comparable to standard univariate voxel-wise analysis with smoothing. As expected, the stimuli (all modulated shapes averaged) evoked clear clusters of activity in all mapped visual areas (Fig 2A). Modulated shapes evoked slightly larger activity than circular shapes in all visual areas (Fig 2B), but the difference was statistically significant only in areas V3v, VO1, V3d, and V3AB (Table 1A). The difference in activation is probably due to adaptation (circles were presented more often) or contrast energy (circles had shortest perimeter). The response magnitude varied significantly across areas (Table 1B) and largest responses for both circles and modulated shapes were found in areas V3d, hV4, VO1, V3AB, LO and TO (Fig 2B). Thus our stimuli well activated areas known to be important for shape processing. The amount of activation slightly increased as a function of local curvature, but the increase was not statistically significant (Table 1B and Fig 2C). The amount of activation also slightly decreased as a function radial frequency, but the decrease was not statistically significant (Table 1B and Fig 2D). There were no significant interactions and the activation across visual areas did not depend on the local curvature or the radial frequency of the stimulus (Table 1B). In sum, the stimuli evoked clear responses across visual areas, but the amount of signal change did not depend on the stimulus parameters.

**Table 1:** Statistical tests.

		**Difference between**	**Area**	**t-test**	**p**		**Fig**
**A**	Signal Changes	Modulated Shapes vs. Circles	V1	t(10) = 1.497	p = .085		2B
			V2v	t(10) = 1.411	p = .084		2B
			V2d	t(10) = 1.741	p = .112		2B
			V3v	t(10) = 2.073	p = .032*		2B
			V3d	t(10) = 2.334	p = .021*		2B
			hV4	t(10) = 1.367	p = .105		2B
			VO1	t(10) = 2.990	p = .007**		2B
			V3AB	t(10) = 2.334	p = .021*		2B
			IPS0	t(10) = 1.259	p = .118		2B
			LO	t(10) = 1.452	p = .088		2B
			TO	t(10) = 1.368	p = .105		2B
			V1	t(10) = 1.497	p = .085		2B
		**Effect of**		**ANOVA**	**p**	**Effect size**	**Fig**
**B**	Signal Changes	Visual Area		F(3.4,33.7) = 4.717	p = .006**	$\eta_{p}^{2}$ = 0.32^¤^	2
		Local Curvature		F(2.5, 25.2) = 0.156	p = .898	$\eta_{p}^{2}$ = 0.02	2C
		Radial Frequency		F(2.5,24.9) = 0.680	p = .547	$\eta_{p}^{2}$ = 0.06	2D
		Interaction: Area x Curvature		F(5.1,50.7) = 1.336	p = .264	$\eta_{p}^{2}$ = 0.12	2C
		Interaction: Area x Radial Frequency		F(5.8,58.2) = 1.101	p = .368	$\eta_{p}^{2}$ = 0.10	2D
		**Effect of**	**Model**	**ANOVA**	**p**	**Effect size**	**Fig**
**C**	Correlation	Hemishpere	RFC	F(1,10) = 0.561	p = .471	$\eta_{p}^{2}$ = 0.05	5A
		Visual area	RFC	F(3.2, 32.0) = 3.719	p = .019*	$\eta_{p}^{2}$ = 0.27^¤^	5A and 5B
		Interaction: Hemisphere x Area	RFC	F(3.6,36.2) = 0.404	p = .786	$\eta_{p}^{2}$ = 0.04	5A
		Visual area	SF	F(3.2,32.0) = 5.799	p = .002**	$\eta_{p}^{2}$ = 0.37^¤^	5B
		Visual area	Energy	F(4.0,39.5) = 3.737	p = .012*	$\eta_{p}^{2}$ = 0.27^¤^	5B
		Visual area	Amplitude	F(3.0,30.5) = 0.383	p = .769	$\eta_{p}^{2}$ = 0.04	5B
		Visual area	Concave	F(2.8,28.4) = 0.803	p = .497	$\eta_{p}^{2}$ = 0.07	5B
		Visual area	Convex	F(4.0,40.4) = 2.148	p = .092	$\eta_{p}^{2}$ = 0.18	5B
		**RFC model, higher than zero**	**Area**	**t-test**	**p**		**Fig**
**D**	Correlation	Left Hemisphere	V2d	t(10) = 2.207	p = .026*		5A
			V3d	t(10) = 2.436	p = .018*		5A
			V3AB	t(10) = 2.878	p = .008**		5A
			IPS0	t(10) = 2.672	p = .012*		5A
		Right Hemisphere	V2d	t(10) = 1.896	p = .044*		5A
			V3d	t(10) = 2.001	p = .037*		5A
			V3AB	t(10) = 2.038	p = .035*		5A
			IPS0	t(10) = 1.865	p = .046*		5A
		**Difference between**	**Model**	**t-test**	**p**		**Fig**
**E**	Correlation	V2d/V3d vs. V2v/V3v	RFC	t(10) = 2.205	p = .05*		5C
		V3AB/IPS0 vs. hV4/VO1	RFC	t(10) = 3.395	p = .007**		5C
		V2d/V3d vs. V2v/V3v	SF	t(10) = -0.270	p = .793		5C
		V3AB/IPS0 vs. hV4/VO1	SF	t(10) = 2.057	p = .067		5C
		V2d/V3d vs. V2v/V3v	Convex	t(10) = 2.085	p = .064		5C
		V3AB/IPS0 vs. hV4/VO1	Convex	t(10) = 2.126	p = .059		5C
		RFC vs. SF model		t(10) = 2.045	p = .034*		5C
		**Effect of**	**Model**	**ANOVA**	**p**	**Effect size**	
**F**	Regression, R^2^	Visual area	RFC	F(2.2, 22.3) = 3.718	p = .036*	$\eta_{p}^{2}$ = 0.27^¤^	
		Visual area	Convex	F(2.3,23.2) = 0.899	p = .434	$\eta_{p}^{2}$ = 0.08	

*p < .05

**p < .01

^¤^large effect size

### Representational similarity analysis of the BOLD signal

**Fig 3:**
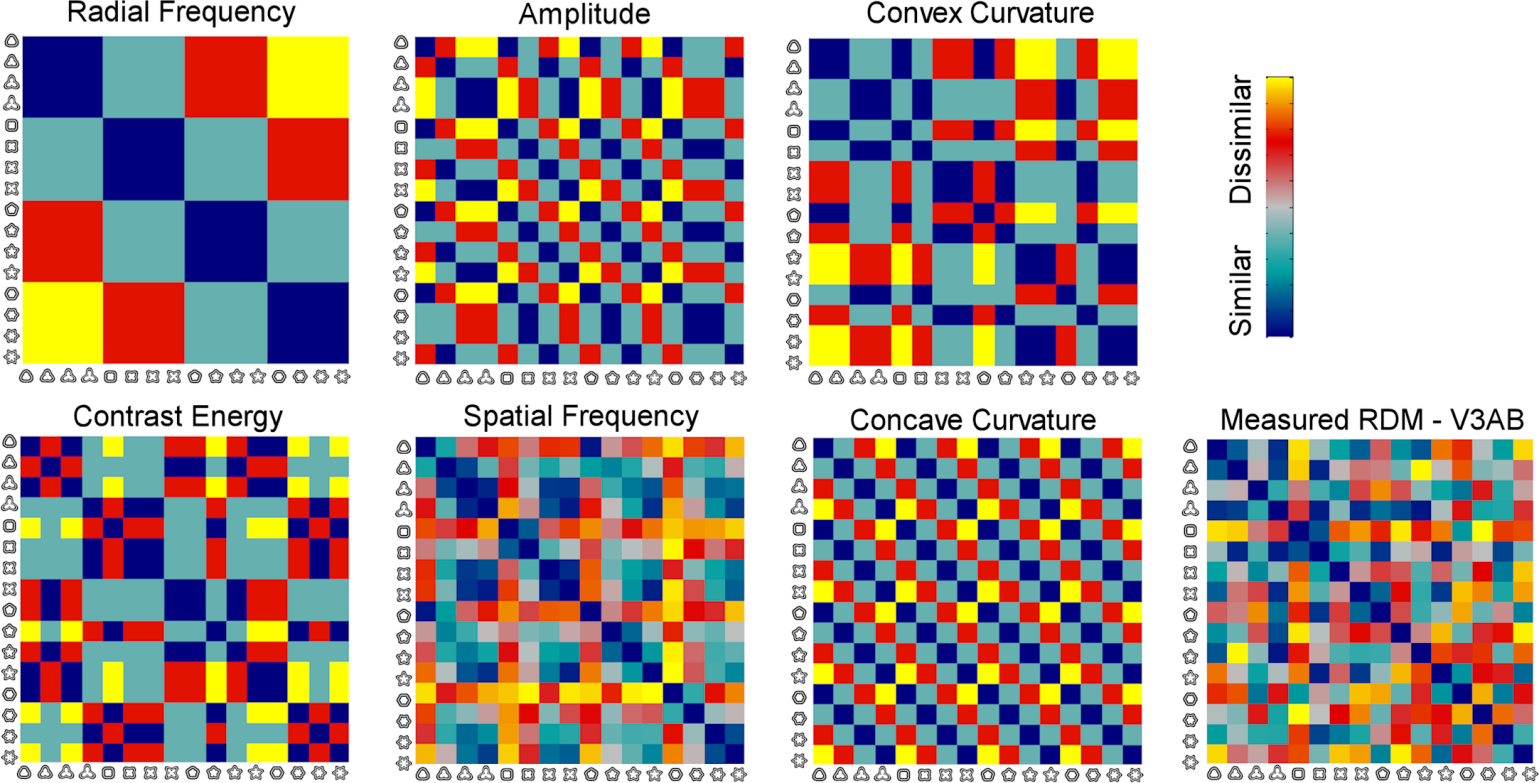
Model and measured RDMs. The RDMs describe the dissimilarity of the response patterns across different shapes. Five models were constructed based on the classification of the stimuli to four classes (Table 2) and one model was constructed by cross-correlating stimulus SF spectrum. Model RDMs for Radial Frequency, Amplitude, Convex Curvature, Contrast Energy, Spatial Frequency Spectrum, and Concave Curvature, and one example of the measured RDM from visual area V3AB. See S1 Fig for examples of measured RDMs in all areas.

Next we investigated if any of the areas represented specific contour shape information. We tested whether the response profiles in any visual area resembled response profiles predicted on the basis of different stimulus parameters: radial frequency, concave curvature, convex curvature, and modulation amplitude. Within each searchlight we calculated a representational dissimilarity matrix (RDM) by cross-correlating the response patterns for the 16 modulated shapes, and compared the measured RDMs to model RDMs based on shape parameters (Fig 3). Visually comparing the measured RDMs (S1 Fig) with the model RDMs (Fig 3) does not reveal any model superior to other models. Visual inspection suggests, however, that there are differences across visual areas and non-random structures in the measured RDMs (S1 Fig) that might be explained by the stimulus parameters.

**Fig 4:**
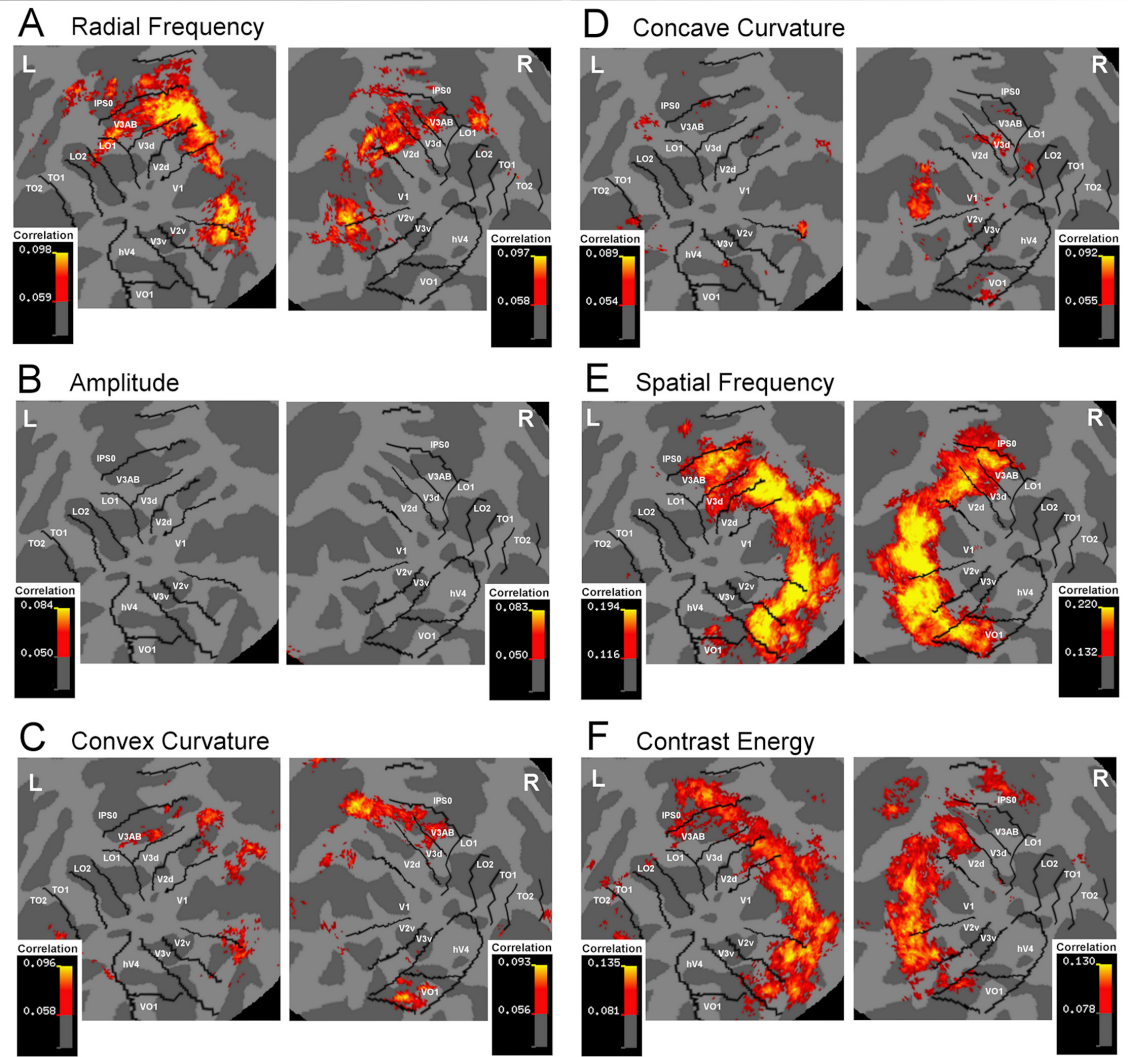
Response profile maps. Correlation maps for Radial Frequency (**A**), Amplitude (**B**), Convex Curvature (**C**), Concave Curvature (**D**), Spatial Frequency spectrum (**E**), and Contrast Energy (**F**). Within the searchlight, correlation with model RDMs was calculated for each voxel. Average correlations, larger than three standard deviations above the mean, across participants are shown on the flattened Freesurfer average surface. Left and right hemispheres on the left and right sides, respectively.

Next, the similarity between the measured and model RDMs was quantified by calculating the correlation between the measured RDMs and model RDMs within the spherical searchlights, and averaged across participants. Fig 4 shows these correlation maps in visual cortex for each model RDM. In general, the response profile maps (Fig 4) resembled the activity maps (Fig 2A), that is, the strongest correlations between brain and model RDMs were found across visual cortices approximately at the same retinotopic locations as the highest activity. However, LO2, TO1 and TO2, while robustly activated in the univariate analysis, were not captured by any of the models in the multivariate analysis.

The highest correlations (>three standard deviations above the mean) between the measured RDM and the RFC-model were found in visual areas V1, V2v, V2d, V3d, V3AB, IPS0, and some voxels in area LO1 (Fig 4A) suggesting that the activation patterns in these areas carry information about the RFC of the stimulus contour. For the amplitude of the shape modulation, no clear peaks in correlation were found in any visual area (Fig 4B). For convex curvature, clusters of high correlations were found in areas V1, V2v, V3AB, and in VO1 in right hemisphere (Fig 4C), and for concave curvature, high correlations were found in few voxels in V1 (Fig 4D).

We also calculated correlation maps for spatial frequency and contrast energy. As the radial frequency of the contour and the amplitude of the modulation are increased, the SF-spectrum of the stimulus shifts slightly to higher frequencies. The amplitude modulates the SF-spectrum more than the radial frequency. As expected, the pattern of activity in areas V1-V3, V3AB, IPS0, and V01 strongly correlated with the SF-spectrum of the stimuli (Fig 4D). Similarly the measured RDMs correlated with contrast energy in areas V1-V3, V3AB, and IPS0 (Fig 4E). Thus, the low-level visual factors—contrast energy and spatial frequency—predicted the dissimilarity of the response patterns non-selectively across visual cortex.

### Visual area analysis

To further quantify the differences between response profiles across visual areas, we conducted a ROI analysis based on the probability atlas of visual areas [33]. In the ROI analysis, areas LO1 and LO2 that are a part of LOC [18], as well as areas TO1 and TO2, were combined. Further, we used the univariate activity maps (Fig 2A) as functional localizer, that is, for each individual we included only voxels that were clearly activated by the stimuli (t-value > 4.0). Separate repeated measures ANOVAs were conducted for every model.

**Fig 5:**
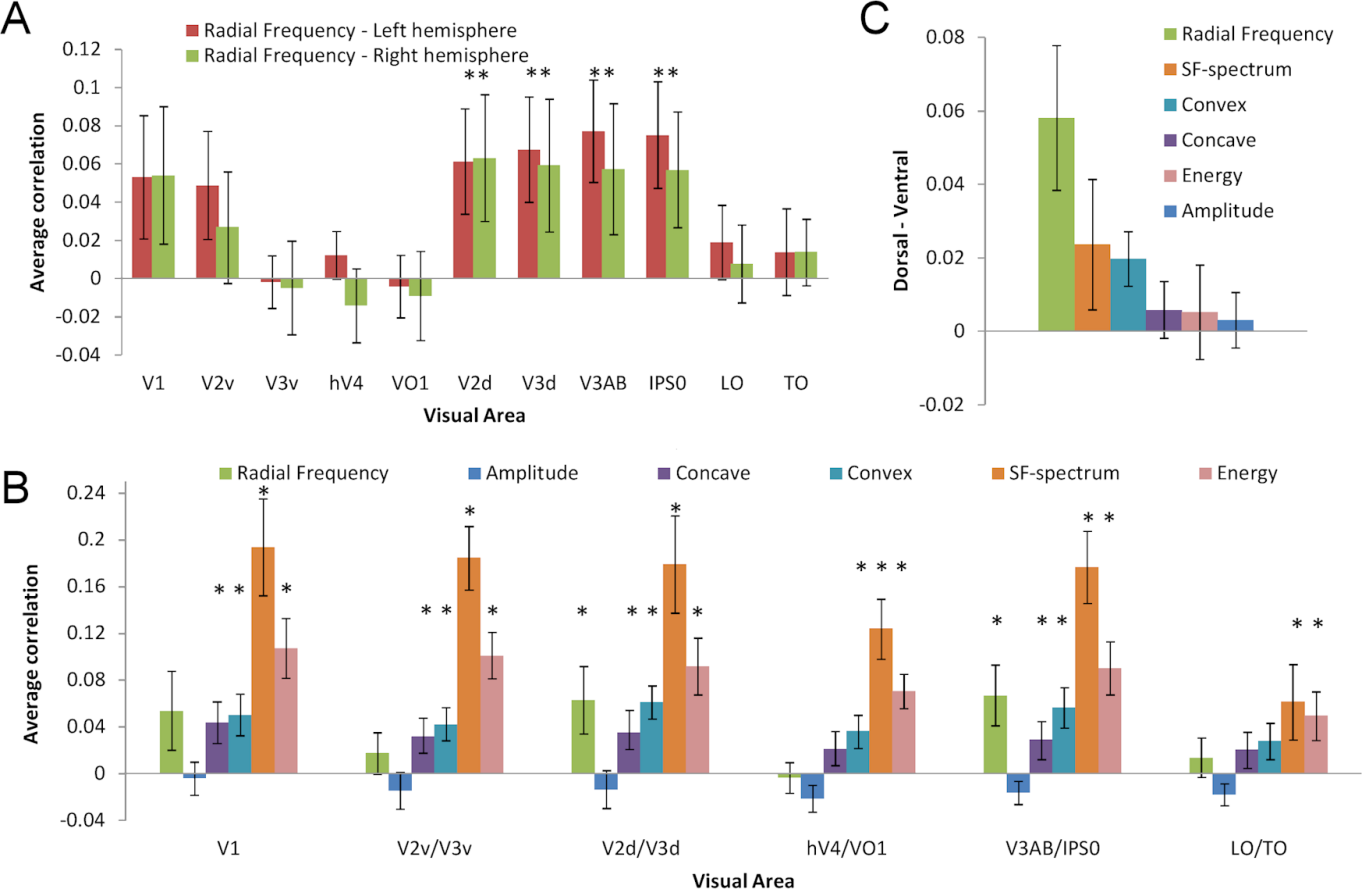
Visual area analysis. **A**) Average correlations with Radial Frequency model for different visual areas in the left and right hemispheres. **B**) Average correlations with all models. Hemispheres have been averaged. Error bars depict standard error of mean across participants. **C**) Dorsal-Ventral difference. Average correlation difference between dorsal (V2d, V3d, V3AB, IPS0) and ventral (V2v, V3v, hV3, VO1) visual areas for model RDMs. *p < .05 (t-test).

The average correlation of the measured pattern and RFC-model did not differ across the left and right hemispheres (Table 1C and Fig 5A). However, the average correlation with RFC-model varied significantly across visual areas (Table 1C and Fig 5A). The highest correlations (significantly above zero, p < .05, t-test) between the measured RDM and RFC model RDM were in areas V2d, V3d, V3AB and IPS0, in both the left and the right hemispheres (Table 1D and Fig 5A). That is, in these visual areas the measured response patterns carried information about the radial frequency of the shape stimuli. The correlations in areas V1 and V2v were not significantly above zero. In areas V3v, hV4, VO1, LO1/LO2 and TO1/TO2, the average correlation with RFC-model was close to zero (Fig 5A). No significant interaction between the hemisphere and visual area (Table 1C) was found for RFC model suggesting that radial frequency is similarly represented in same visual areas in both hemispheres. Thus, the statistical analyses confirm the selective spread of pattern correlations for radial frequency only for certain visual areas, as was shown in the Fig 4A.

The effect of hemisphere was not significant for any model indicating robustness of the result. The correlations varied significantly across visual areas also for SF and contrast energy, but not for amplitude, concave curvature or convex curvature (Table 1C). The effect of SF and contrast energy was expected since these are the primary factors that drive the low-level neural responses. However, the modulation amplitude as such and the direction of local curvature seem not to be represented specifically in any certain visual area. The interaction between the hemisphere and visual area was not significant for any model, showing that response patterns were similar in the same visual areas in both hemispheres.

Since no significant effects of hemisphere were found, the correlations for each model RDM were averaged across hemispheres, and tested with t-tests (significantly above zero, p < .05) to statistically confirm the spread of correlations across visual areas (shown in Fig 4). We used probabilistic atlas to localize the visual areas and this might produce some uncertainty to classifying areas close to each other. Therefore, nearby visual areas were averaged as follows: V1, V2d/V3d, V3AB/IPS0, V2v/V3v, hV4/VO1, and LO/TO. The average correlation of the measured RDM with RFC model varied across areas and was significantly above zero in V2d/V3d and V3AB/IPS0 (Fig 5B). The average correlation with Amplitude model RDMs was constantly low and not significantly higher than zero in any of these areas. The average correlation to other models remained high across most of the areas: correlation with SF and energy models was significantly above zero in all areas, correlation with concave model was significantly above zero in all areas except LO/TO, and correlation with convex models was significantly above zero in all areas except hV4/VO1 and LO/TO (Fig 5B).

The clearest area specificity was found for radial frequency. This selectivity was further quantified by comparing (paired sample t-test) the average correlation in dorsal and ventral areas. Statistically significant difference between the ventral and dorsal part of areas V2 and V3, as well as between areas V3AB/IPS0 and hV4/VO1 was found only for RFC, but not for other models (Table 1E). The average difference across all dorsal (V2d/V3d/V3AB/IPS0) and ventral (V2v/V3v, hV4/VO1) areas was largest for radial frequency (Fig 5C), and this difference was significantly higher than for SF (Table 1E).

### Searchlight RSA regression analysis

To further test the independent role of different stimulus parameters, a multiple linear regression analysis within each searchlight was conducted. The regression model contained all six model RDMs: radial frequency, amplitude, convex and concave curvature, contrast energy, and SF spectrum. The average R^2^ was 0.11, and varied between 0.09 and 0.13 depending on the visual area. Thus, all the models explained 11% of the total variability within the searchlights. Next we conducted regression analysis with leave-one-out method and calculated the change of R^2^ values relative to the full model. Removing the SF model from the regressors decreased R^2^ values ca. 40%. For other models, the decrease was 7–16%. Thus most of the explained variability was due to the SF model. This was expected since highest correlations were found for the SF model, and the SF model contains the same information of the stimulus variability as the other models combined. Next we replicated the regression analyses without the SF model. The average R^2^ of the full model decreased to 0.06. In the leave-one-out analysis, largest decrease of R^2^ values were found for convex curvature (29%) and radial frequency (23%). However, only for radial frequency the relative decrease of R^2^ values varied significantly across areas (Table 1F) and was more prominent in dorsal (V2d/V3d and V3AB/IPS0) than in ventral (V2v/V3v and hV4/VO1) areas. Thus, a similar specificity for RFC across visual areas was found in the regression analysis as in the correlation analysis.

## Discussion

Multivariate representational similarity analysis revealed that the RFC of the contour is represented in human visual areas V2d, V3d, V3AB and IPS0. Surprisingly, the areas hV4 and LO1/LO2, known to be important in global shape processing [15, 19, 20], while responding to the stimuli, did not show pattern selectivity for radial frequency. Low-level visual properties—SF spectrum and contrast energy–did not explain our results, since these parameters did not show similar specificity across visual areas as the radial frequency. Our results provide evidence for radial frequency based representations which could be used in contour shape processing, and in particular, we suggest that RFC representations are a mid-level link between local contour analysis in V1 and more comprehensive global shape analysis in areas such as hV4 and LOC. Alternatively, the radial frequency information in the early and intermediate areas may be utilised more directly in the higher level object sensitive areas with no additional middle steps.

Most previous fMRI studies on contour shape perception have compared BOLD-responses to different shapes, i.e., circular vs. parallel gratings [14] or global circular shapes vs. only local curvatures [15]. In the former study hV4 and FFA showed selectivity for concentric shapes, and in the latter study visual areas V3, VP and hV4 showed strongest responses to circularity. Consistent with these studies, our modulated shapes evoked larger responses than circular shapes in univariate analysis of mean signal change, i.e. in the overall fMRI response. The response magnitude did not, however, depend on the local curvature or radial frequency. In order to investigate the role of different stimulus parameters on shape representations, we investigated the multivariate similarity structure [31, 34] of the activity patterns evoked by parametric variation of the contour shapes. The multi-voxel pattern analysis is more sensitive than direct comparison of average responses within the visual area, because the multidimensional pattern of BOLD-responses across voxels contains more information about the response than the averaged one-dimensional measure. Further, the searchlight based approach [32] makes no assumptions where the stimulus specific activation patterns should be found. Our results provide further evidence that radial frequency is used in the contour shape analysis in the visual cortex. Furthermore, our results suggest that RFC based representations are located in visual areas V2d, V3d, V3AB and IPS0. For areas hV4 and LO1/LO2 we did not find evidence for RFC based representations.

Integration of local visual features to contours likely involves visual areas at different processing levels [35, 36]. The representations of contour convexity and concavity, as well as the representations of global shape are likely located in visual areas V3AB, hV4 and LOC [14, 15, 18, 20, 37–40]. In agreement with these studies, largest univariate responses in our study included areas V3AB, hV4 and LO1/LO2. However, the multivariate patterns were different between these areas. Our results suggest that the global closed-shape representations in V3AB are based on the radial frequency of the contour, but we did not find similar RFC based activity patterns in hV4 or LO1/LO2.

The lack of pattern specificity in hV4 and LOC was not due to our stimuli as such, since they robustly activated also these areas. One possibility is that shape representations or neurons encoding shapes in hV4 are so close to each other that voxel-level activity patterns measured with fMRI cannot discriminate them or the MVPA methods are not sensitive enough. This would suggest different structure for V3AB and hV4 neurons/representations since we did find significant voxel-level pattern correlations in V3AB. In primates, cell density [41] and microvascular density [42] vary across cortical areas which might affect BOLD-responses, and thus this is a possible explanation for the difference between the areas we found. Future studies could aim to image the RFC representations in these areas with smaller voxel size or higher spatial resolution using high-field fMRI. Second possible explanation for the difference between the visual areas is that fMRI might be particularly sensitive for feedback [43, 44]. Multivariate pattern reflects data distributed in large part of a functional area, whereas the univariate pattern is sensitive to more local changes. In this scheme the multivariate analysis might better see the feedback effects which typically have much wider distribution than the classical receptive field [45] and this would emphasize the early areas as well as give different distribution in the mid-level areas. Third possibility is that—since the RFC based representations are mainly limited to closed shapes—areas that represent more complex visual objects, such as hV4 and LOC, might simply use some other type of shape encoding.

Instead of individual functional localization of visual areas, we used probability atlas of visual areas measured in a separate study [33]. The average location of early visual areas (e.g., V1-V3) is more accurate than subsequent visual areas (e.g., hV4, LO1/LO2). Hence there might be more locational variability in the activity patterns in hV4 and LO1/LO2 across individuals and this might explain the absence of RFC specific activity patterns. However, we did find significant correlation between measured patterns and model RDMs for SF Spectrum and Contrast Energy also in areas hV4/VO1. In our searchlight analysis the activity patterns are smoothed with the spherical volume of searchlight, and we calculated an average within the ROIs. This analysis controls for small deviations in exact locations of activity patterns. Furthermore, all mapped visual areas, including hV4 and LO1/LO2, were activated by our stimuli and these univariate activation maps were used as functional localizers in the ROI analysis of RDM correlations. This emphasizes voxels across visual areas that were indeed processing our stimuli. Still, some locational uncertainties in our results remain. However, the locational uncertainties are more likely between nearby areas, e.g., between areas V2 and V3, than areas further away, such as between areas V3AB and hV4.

Another limitation of our study is the relatively low correlations found between measured and model RDMs. While the correlations were quite low, the results were systematic across participants, and the effect sizes (of the ANOVAs testing the effect of visual area) were large (Table 1). Instead of the correlation values as such, the structure of correlations across studied events was the main interest in our study. These structures reveal information of the representational geometry that can be compared to predictions based on different models, and we found clear differences between the models based on RFC of the contour and models based on the other stimulus parameters. Further, most of the previous RSA studies have investigated representational similarities across object categories. In contrast, we studied representational similarities of relatively similar shapes within a category, which could also explain the low correlation values we obtained.

The asymmetry between the dorsal and ventral areas for RFC model could be related to the ecologically justified and well known difference between the upper and lower visual fields [46]. Anatomically, there is slightly more cortex representing the lower than the upper visual field in macaque monkey V1 [47], physiologically stronger responses in MEG in humans [48, 49], and better behavioral performance in humans [46, 50]. In line with these earlier findings, our results suggest that radial frequency representations are biased towards cortical areas with lower visual field representations. This finding can be contributed by the relative cortical size of the lower vs. the upper visual field representations, and differences in the represented eccentricities in dorsal and ventral areas. However, we did not find similar asymmetry for contrast energy and spatial frequency. The anisotropy between IPS0/V3AB and hV4/VO1 areas cannot be explained by retinotopy, because all these areas comprise half-field representations, i.e. both the upper and lower visual fields [51]. Consistent with our results, lower visual field advantage was recently demonstrated for perception of RFC shape stimuli whereas no similar asymmetry was found for orientation or curvature discrimination [52].

The radial frequency and amplitude of the modulation determine the shape of an RFC pattern. However, several other parameters vary as the RF and amplitude of the stimulus is varied. Increasing the amplitude and the radial frequency increases the contour length and contrast energy and shifts the SF spectrum to higher SFs. The RDM models based on these low-level visual parameters did correlate with the measured patterns across visual areas but without similar specificity as was found for radial frequency. Thus the results found with the RFC-model are not due to these low level factors but reflect the different activity patterns evoked by the parametric modulation of RFC. For the amplitude of modulation the average correlation was constantly near zero, as expected. The amplitude as such is not a critical parameter for visual shape analysis. Slightly higher correlations were found for convex than concave curvature, but the correlations did not vary much across visual areas for these parameters. Higher correlations for convex curvature might indicate more critical role of convex than concave forms and angles in shape analysis, as previously suggested [4, 13, 28, 40, 53, 54].

In our experiment, all the stimuli were presented in four different orientations. As the orientation of the shape was varied, the shapes activated slightly different retinotopic locations. However, the RSA analysis was conducted for SPM_T_-images in which the shapes in different orientations had been averaged. Thus the role of different retinotopic locations was controlled already in GLM analysis. We did a separate control analysis with a different GLM model (shapes averaged across different amplitudes instead of orientations) and calculated correlation maps for the orientation and the retinotopic locations. As expected, the correlation map for the orientation as such did not reveal any peaks in any visual area. The correlation map for retinotopy revealed clear activity peaks across visual areas and was highly similar to the correlation map for the SF spectrum.

Recently, radial frequency patterns and multi-voxel pattern analyses have been used to study perception of RFC motion trajectories [55, 56]. The motion trajectory of a dot could be decomposed from areas V2, V3 and MT [55, 56]. In contrast, the shape of the static RFC patterns could not be decoded in these areas, but only in posterior parietal areas and in LOC [56]. In contrast, we found RFC specific response profiles in areas V2-V3, and V3AB. There are several differences in our and Gorbet et al. [56] experimental setups and data analysis which likely explain the different results. Most likely our setup was more sensitive to differences between RFCs because we had much higher number of stimulus presentations, and we compared response patterns to all different RFCs simultaneously, instead of comparing only two RFCs at a time [56]. Nevertheless, both our and Gorbet et al. [55, 56] studies agree in that radial frequency is used in the visual shape analysis in areas V2 and V3, but not in area hV4.

The prevailing theoretical view suggests that neural representation of the visual environment is built from a sparse set of basis functions whose combinations constitute the population code for perceptual representations [57, 58]. It is possible that the RFC representations, corresponding to the relatively simple combinations of Gabors, provide a set of mid-level basis functions for shape analysis. Our results provide further support for the idea of radial frequency based representations in shape perception, and suggest that the neural mechanisms that utilize radial frequency are located in the intermediate visual areas V2d, V3d, V3AB and IPS0, but not in areas hV4 or LOC. This result places the radial frequency representations to relatively early position in visual processing, presumably beyond Gabor analysis, but before object identification.

## Materials and Methods

### Ethics statement

The ethics committee of the Hospital District of Helsinki and Uusimaa had approved the experiments (Coordinating ethics committee, Dnro 299/13/03/00/2010). The experiments were conducted according to the declaration of Helsinki and participants gave written informed consent before the measurements.

### Participants

Eleven participants (one female), with normal or corrected-to-normal vision, participated in the study. First and last author participated as subjects; the rest of the participants were naïve to the purpose of the study.

### Stimuli

The stimuli were radial frequency patterns (Fig 1A), which were constructed by sinusoidally modulating (Fig 1C) the radius of a base circle [1]. The spatial profile of the contour was 4^th^ derivative of Gaussian and the peak spatial frequency of the contour was 1.57 c/deg (σ = 0.28 deg). The shapes were composed of four different radial frequencies, amplitudes (Fig 1A) and orientations (Fig 1B). The radial frequencies were 3 (triangle), 4 (quadrilateral), 5 (pentagon) and 6 (hexagon) cycle/perimeter. The minimum and the maximum local curvature of the contour depend on the radial frequency and the amplitude of the shape (equation 4 in [1]). The amplitude of the shapes was varied so that the maximum local curvatures at the peak (point of maximum curvature or convex curvature) and the trough (point of minimum curvature or concave curvature) of the radial modulation (Fig 1D) were roughly equal across different radial frequencies (Table 2). The amplitude varied between 0.0 and 0.46 in proportion to the radius, the maximum concave curvature varied between -0.1 and -4.8 deg^-1^, and the maximum convex curvature varied between 0.6 and 2.3 deg^-1^ (Table 2). Each stimulus was presented in four orientations, which corresponded to polar phases 0, 90, 180 and 270 deg (Fig 1B). In total there were 65 different stimuli (circle + 4 radial frequencies x 4 amplitudes x 4 orientations). The rms-contrast (the standard deviation of the luminance divided by the mean luminance) of the stimuli was 0.17, and same for all the stimuli. The radius of the base circle was 2.86 deg and the stimulus maximum diameter varied from 5.7 (circle) to 8.6 deg (radial frequency four with amplitude of 0.35).

**Table 2:** Stimulus parameters and classifications in model RDMs.

Stimulus	Radial frequency	RFC-model	Concave curvature	Concave model	Convex curvature	Convex model	Amplitude	Amplitude model	Contrast energy	Energy model
1	3	1	-0.09	1	0.61	1	0.12	1	0.37	1
2	3	1	-0.85	2	0.77	1	0.24	3	0.61	3
3	3	1	-2.07	3	0.86	2	0.35	4	0.38	1
4	3	1	-4.61	4	0.92	2	0.47	4	0.54	2
5	4	2	-0.22	1	0.74	1	0.09	1	0.66	4
6	4	2	-1.07	2	1.02	2	0.18	2	0.54	2
7	4	2	-2.18	3	1.19	3	0.26	3	0.45	2
8	4	2	-4.10	4	1.33	3	0.35	4	0.57	3
9	5	3	-0.33	1	0.86	1	0.07	1	0.6	3
10	5	3	-1.25	2	1.25	3	0.14	2	0.68	4
11	5	3	-2.50	3	1.54	4	0.21	3	0.61	3
12	5	3	-4.24	4	1.77	4	0.28	4	0.29	1
13	6	4	-0.48	1	1.00	2	0.06	1	0.33	1
14	6	4	-1.55	2	1.52	3	0.12	2	0.71	4
15	6	4	-2.94	3	1.92	4	0.18	2	0.43	2
16	6	4	-4.77	4	2.25	4	0.24	3	0.82	4

Four different radial frequencies (3–6) and four different amplitudes were used. The amplitudes of the shapes were selected so that the local curvature was roughly equal across different RFCs. Concave curvature refers to the local curvature at the troughs of the modulation function (Fig 1D) and convex curvature refers to the local curvature at the peaks of the modulation function (Fig 1D). Stimulus parameter based model RDMs (Fig 3) were based on classification of stimuli to four different classes.

### fMRI data acquisition

The fMRI data were acquired with a Siemens MAGNETOM Skyra 3 T scanner (Siemens Healthcare, Erlangen, Germany) equipped with a 30- or 32-channel receive only head coil. Each measurement session started with a fast structural MR image with a 3D T1-weighted sequence (in-plane resolution 1.8x1.8 mm, and 1.5 mm slice thickness). Then eight experimental runs were measured using a gradient-echo echo planar imaging sequence (TR = 1800 ms, TE = 30 ms, flip angle = 60 deg, 64x64 acquisition matrix, FOV = 20 cm, 23 slices, 3.0 mm slice thickness, resulting in 3.1 x 3.1 x 3.0 mm voxel size).

The fMRI data were analyzed with SPM8 Matlab toolbox [59] and Freesurfer [60] software packages. The preprocessing comprised first the correction for the acquisition order of the functional images and then for the head motion.

### Procedure

Circles and 64 different contour shapes were presented in event-related design. In each run all shapes were presented once, except circular stimulus, which was presented 16 times. In addition, 20 rest trials were included. In total, one run consisted of 100 events (20 rests + 16 circles + 64 modulated shapes). The duration of each stimulus was 300 ms and the duration of each trial 2.4 seconds. Hence, the length of the run was 240 s (100*2.4s, corresponding to 135 volumes). Each run started with eight volumes, which were discarded from the analysis to reach stable magnetization. Stimuli were presented with abrupt on/offset. In total, eight runs were measured resulting in 800 stimulus presentations. The order of stimuli in each run was optimally randomized [61] and the order of runs was randomized across participants. Each radial frequency was presented 128 times (4 (amplitudes) x 4 (orientations) x 8 runs).

The shapes were presented at 10 deg eccentricity (at 43-cm viewing distance), one (same) shape in each quadrant in order to have separable upper/lower and left/right visual field responses in early visual areas. The participants performed a demanding RSVP attention task at the fixation [18]. Five different letters (Z,L,N,T, and X; Arial-font) were rapidly presented at fixation (150 ms/letter). The letter series contained 1–4 ‘X’-letters and the participants’ task was to count the ‘X’s and report the number of ‘X’s during a 1 s break in every 5.4 seconds. The letter task was used to control for participants attention. The letter task, instead of shape discrimination task, was also used to avoid ceiling effects in behavioral performance since the contour shapes were supra-threshold, far exceeding the contrast sensitivity and discrimination thresholds of these patterns.

### Data analysis

The general linear model (GLM) analysis included a design matrix where the data were modelled with 17 effects of interest (1 for circle, 16 for different modulated shapes) and 8 nuisance regressors (1 for RSVP-letters, 1 for responses to attention task, and 6 for head motion parameters). Data were high-pass filtered at 1/200 Hz. SPM_T_-images were calculated for the 17 stimulus-related regressors, and corresponding BOLD signal changes for each voxel by dividing the parameter estimates with mean response. In the experiment each shape was presented in four different orientations. The orientation was omitted from the analysis and each shape was modelled with one regressor. Separate control analysis confirmed that orientation as such did not have significant effect on the measured activity patterns.

Next, we applied several searchlight analyses [32]. The radius of the spherical searchlight was 3 voxels, resulting in an average search volume of 100 voxels (similar results were obtained with smaller (70 voxels) and larger (270 voxels) searchlight volumes). First searchlight analysis was conducted to find visual areas that were activated by the contour stimuli. For a univariate activity map, comprising ca. 30000 voxels, we first centered the searchlight volume at each voxel, and then calculated within the volume the T-value of average signal change across the different shapes, i.e., stimulus-related regressors. The circle shape was omitted from the analysis because it was presented more often than other shapes, and it evoked weaker responses than modulated shapes. The same analysis was repeated for each participant. The result essentially corresponds to classical activity map but with smoothing by the searchlight volume.

In the subsequent searchlight analyses, we calculated a representational dissimilarity matrix (RDM; [31]) within each searchlight. The RDM comprised 1—Pearson correlation between the response patterns for the 16 visual shapes (the circle shape was again omitted from the analysis). The logic of the searchlight analysis is straightforward: If the multi-voxel response pattern, contained within the spherical searchlight, carries information about a parameter, such as the stimulus shape, then there should be high correlation between voxel response patterns for similar shapes and low correlation for dissimilar shapes. To find out what information the visual areas were representing, the measured RDMs within the searchlights were compared to different model RDMs [31, 62]. The comparison was quantified as Spearman correlation between the measured and model RDMs. Since the RDMs are symmetric over the diagonal, only the lower triangular parts of the matrices were used in the comparison. The searchlight RSA analysis were conducted using the RSA toolbox [63].

### Model RDMs

The RDM models for Radial Frequency Components (RCF), Amplitude, Concave and Convex Curvature were constructed by classifying the stimuli according to the parameter of interest (Fig 3; Table 2). In the RDM for RFC, for example, the stimuli are classified according to the radial frequency of the stimulus (RFC 3–6), and nearby radial frequencies are expected to be represented more similar than more distant radial frequencies. Thus, if the RFC of the shape is represented in any visual area, then the activity patterns for the same RFCs are expected to be more similar (higher correlation) than the activity patterns for different RFCs. A similar logic applies to other features of the stimuli. Separate models for concave and convex local curvature were used since previous studies have suggested different role of concavities and convexities in shape perception [53] and in fMRI [40]. To control for low-level visual feature similarity between the stimuli, two additional RDM models were calculated for Contrast Energy and Spatial Frequency (SF) spectrum of the stimuli (Fig 3). For the Contrast Energy model, total contrast energy (sum of squared pixel contrast values) for each stimulus was calculated and classified to create the model RDMs (Table 2). For the SF model, SF spectrum of each stimulus was calculated and cross-correlated to create model RDM.

In total 7 searchlight maps (activity map + 6 RSA correlation maps) were calculated for each participant. The individual searchlight maps were projected on the Freesurfer average cortical surface, and averaged across participants. The visual areas were identified from the Freesurfer average surface on the basis of probability atlas of visual areas measured in the separate study [33]. The statistical significance of the average signal changes and correlations across participants were tested with repeated measures ANOVAs and t-tests. Greenhouse-Geisser correction was used in ANOVAs when Mauchly's test of sphericity was significant.

## Supporting Information

S1 Fig Measured RDMs. **A)** Measured RDMs from different visual areas averaged across participants. Visual evaluation of measured matrices does not reveal any clear structure comparable to model RDMs (Fig 3) or clear differences between visual areas. **B)** Measured RDMs and RFC model averaged to 4×4 matrices. These averaged RDMs were most similar with the RFC model RDM in areas V3d (r=.32), IPS0 (r=.42) and LO (r=.42), but the correlations were not statistically significant (permutation test).
(PDF)
