# Supplementary material for: Radial Frequency Analysis of Contour Shapes in the Visual Cortex
Source: PLoS Comput Biol. 2016 Feb 11;12(2):e1004719. doi: 10.1371/journal.pcbi.1004719 (PMC4750910; doi:10.1371/journal.pcbi.1004719)
Supplement: S1 Fig — A) Measured RDMs from different visual areas averaged across participants. Visual evaluation of measured matrices does not reveal any clear structure comparable to model RDMs (Fig 3) or clear differences between visual areas. B) Measured RDMs and RFC model averaged to 4×4 matrices. These averaged RDMs were most similar with the RFC model RDM in areas V3d (r=.32), IPS0 (r=.42) and LO (r=.42), but the correlations were not statistically significant (permutation test). (PDF) [file pcbi.1004719.s001.pdf]

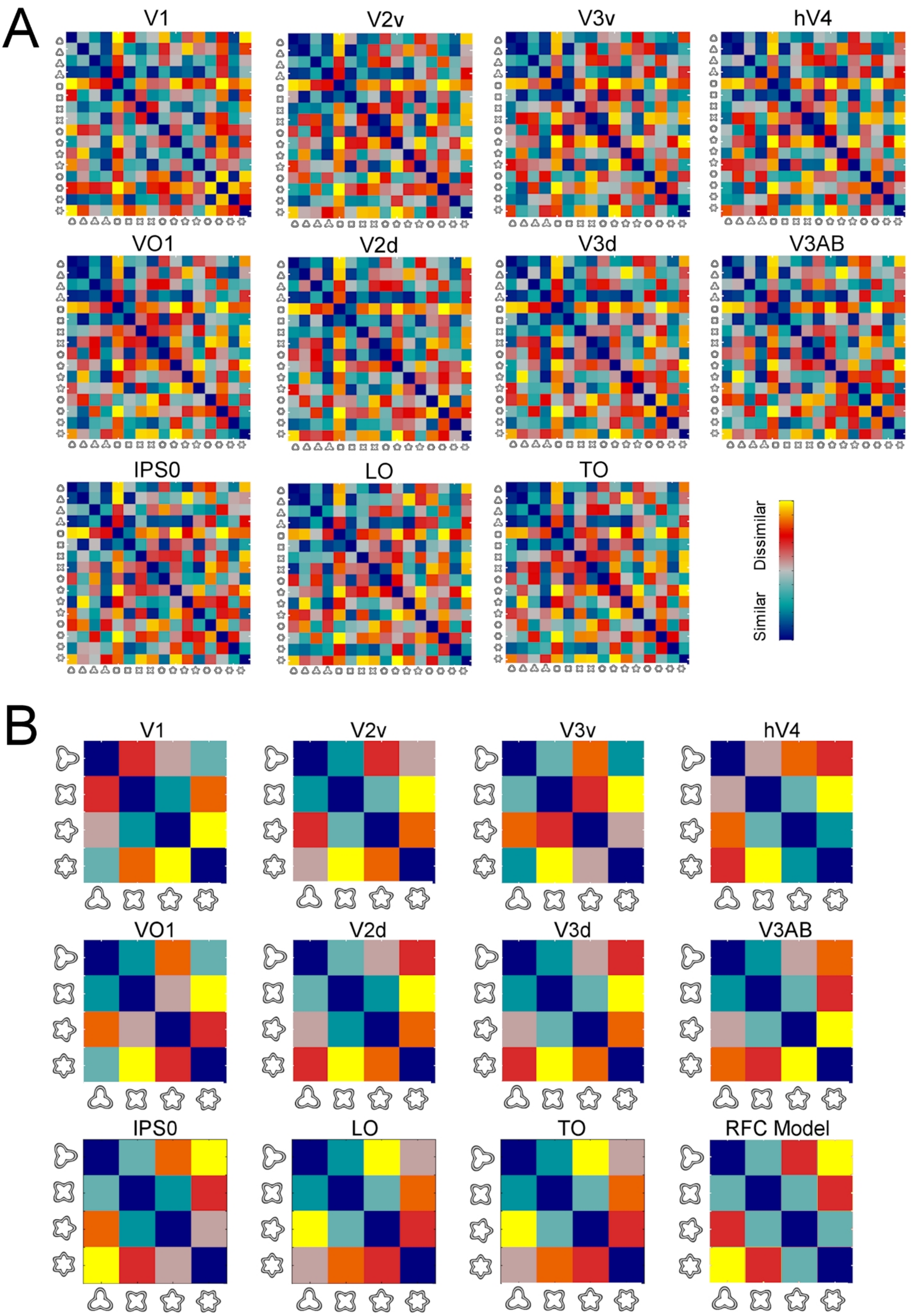

S1 Figure. Measured RDMs. *A) Measured RDMs from different visual areas averaged across participants. Visual evaluation of measured matrices does not reveal any clear structure comparable to model RDMs (Fig. 3) or clear differences between visual areas. B) Measured RDMs and RFC model averaged to 4x4 matrices. These averaged RDMs were most similar with the RFC model RDM in areas V3d (r=.32), IPS0 (r=.42) and LO (r=.42), but the correlations were not statistically significant (permutation test).*
